# Correlates of protection against symptomatic SARS-CoV-2 in vaccinated children

**DOI:** 10.1038/s41591-024-02962-3

**Published:** 2024-04-30

**Authors:** Youjia Zhong, Alicia Y. H. Kang, Carina J. X. Tay, Hui’ En Li, Nurul Elyana, Chee Wah Tan, Wee Chee Yap, Joey M. E. Lim, Nina Le Bert, Kuan Rong Chan, Eugenia Z. Ong, Jenny G. Low, Lynette P. Shek, Elizabeth Huiwen Tham, Eng Eong Ooi

**Affiliations:** 1 Department of Paediatrics, Yong Loo Lin School of Medicine, National University of Singapore (NUS), Singapore, Singapore; 2 Programme in Emerging Infectious Diseases, Duke-NUS Medical School, Singapore, Singapore; 3 Khoo Teck Puat-National University Children’s Medical Institute, National University Health System (NUHS), Singapore, Singapore; 4 Infectious Diseases Translational Research Programme, Department of Microbiology and Immunology, Yong Loo Lin School of Medicine, National University of Singapore, Singapore, Singapore; 5 Viral Research and Experimental Medicine Centre, SingHealth Duke-NUS Academic Medical Centre, Singapore, Singapore; 6 Department of Infectious Diseases, Singapore General Hospital, Singapore, Singapore; 7 Department of Clinical Translational Research, Singapore General Hospital, Singapore, Singapore

**Keywords:** Adaptive immunity, Infectious diseases

## Abstract

The paucity of information on longevity of vaccine-induced immune responses and uncertainty of the correlates of protection hinder the development of evidence-based COVID-19 vaccination policies for new birth cohorts. Here, to address these knowledge gaps, we conducted a cohort study of healthy 5–12-year-olds vaccinated with BNT162b2. We serially measured binding and neutralizing antibody titers (nAbs), spike-specific memory B cell (MBC) and spike-reactive T cell responses over 1 year. We found that children mounted antibody, MBC and T cell responses after two doses of BNT162b2, with higher antibody and T cell responses than adults 6 months after vaccination. A booster (third) dose only improved antibody titers without impacting MBC and T cell responses. Among children with hybrid immunity, nAbs and T cell responses were highest in those infected after two vaccine doses. Binding IgG titers, MBC and T cell responses were predictive, with T cells being the most important predictor of protection against symptomatic infection before hybrid immunity; nAbs only correlated with protection after hybrid immunity. The stable MBC and T cell responses over time suggest sustained protection against symptomatic SARS-CoV-2 infection, even when nAbs wane. Booster vaccinations do not confer additional immunological protection to healthy children.

## Abstract (web summary)

The dynamics and durability of immune responses associated with protection against symptomatic infection in children offer insights to guide vaccination policies in pediatric populations.

## Main

Since the emergence of SARS-CoV-2 as a human-to-human transmitted pathogen in 2020, COVID-19 is now endemic globally. The burden of severe COVID-19 has been mitigated by widespread immunity from vaccination and infection; however, the endemicity and expected cyclical epidemics of COVID-19 will continue unabated due to limited immune-mediated protection against virus transmission^1^. Primary vaccination of future birth cohorts of children will likely continue for the foreseeable future to upkeep population immunity against SARS-CoV-2.

COVID-19 vaccination was implemented at the height of the pandemic, at a time when transmission rates were high, so most vaccinees acquired natural infection shortly after vaccination. Therefore, there is a paucity of information on the longevity of vaccine-induced adaptive immunity. Moreover, neutralizing antibodies against SARS-CoV-2 were widely used to infer protection against COVID-19 even though adaptive immunity was mostly, and sometimes even only, assessed serologically; the protective capacity of the cellular components of adaptive immune responses remains ill-defined^2–6^. A detailed knowledge of these responses would be critical not only to inform global health policies on COVID-19 prevention, but also to shape vaccine development and response to future viral pandemics.

To address these knowledge gaps, we conducted a cohort study that examined the longevity of SARS-CoV-2 spike (S)-binding IgG, nAbs, MBCs and T cells produced following messenger RNA (mRNA) vaccination in SARS-CoV-2-naive children, as well as the immunological effect of a booster (third) dose. As transmission of the Omicron variant in Singapore coincided with this study, we also differentiated hybrid immunity produced from different combinations of vaccination and infection. Finally, we utilized our longitudinal sampling and the endemic transmission of Omicron SARS-CoV-2 to define immune correlates of protection against symptomatic SARS-CoV-2 infection.

## Results

### Participant characteristics

**Fig. 1:**
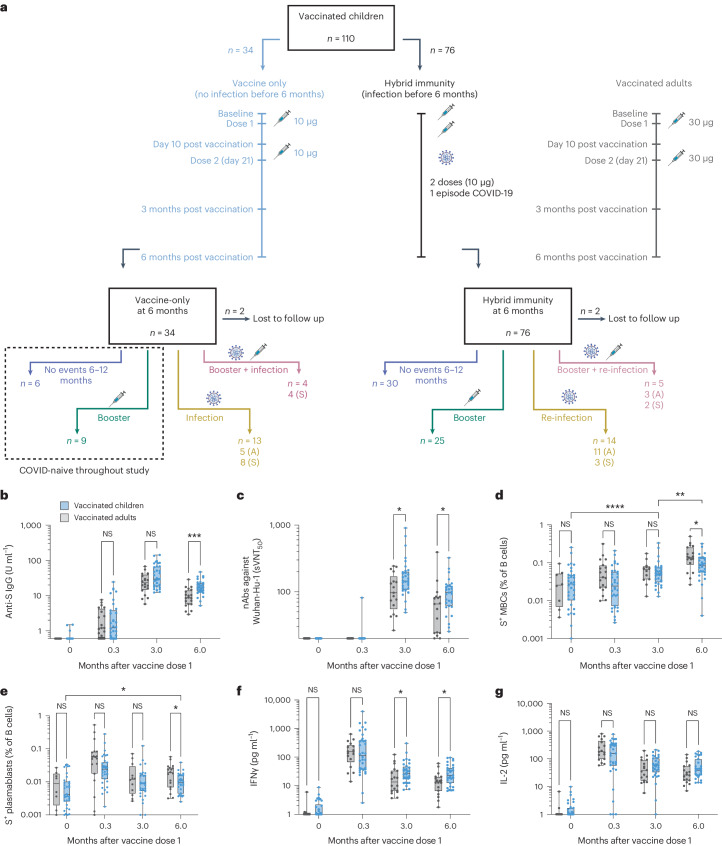
Adaptive immune responses following BNT162b2 vaccination in children aged 5–12 years were comparable or superior to adults despite reduced vaccine dose. **a**, Schematic of study schedule for children in the MARkers of Vaccine Efficacy and Longevity in SARS-CoV-2 (MARVELS) study up to 6 months after vaccination, as well as a flowchart of individuals between 6 and 12 months, divided into children with vaccine-only immunity at 6 months (*n* = 34) and children with hybrid immunity at 6 months (*n* = 76). Children were given two doses of monovalent 10 μg BNT162b2 on day 0 and day 21 of the study. Venous blood was drawn at pre-vaccination baseline, day 10 after dose one, 3 months after vaccination and 6 months after vaccination. A mirror cohort of healthy adult healthcare workers, who were given two doses of monovalent 30 μg BNT162b2, was used as the reference for immunogenicity parameters. Convenience sampling was used for adults, with 18 to 20 individuals per immunogenicity comparison. Children who acquired natural SARS-CoV-2 infection, both symptomatic and asymptomatic, were excluded from comparison to adults. **b**, Anti-S IgG titers at pre-vaccination baseline, day 10, 3 months and 6 months after vaccine dose one. **c**, Antibodies that neutralized 50% of Wuhan-Hu-1 S protein RBD binding to ACE2, as measured using sVNT_50_. **d**, Percentage of S^+^ MBCs out of total B cells. **e**, Percentage of S-specific plasmablasts out of total B cells. **f**,**g**, S-reactive T cell responses measured by post-stimulation IFNγ (**f**) and IL-2 (**g**) levels. For box-and-whisker graphs, the top and bottom boundaries of the boxes indicate the upper and lower quartiles, respectively, the line indicates the median and whiskers represent the range. For all panels, a two-tailed Mann–Whitney *U*-test was used for comparisons between vaccinated children and adults and a Wilcoxon rank test was used for paired comparisons of the same individuals at different time points. NS, not significant, *P ≤ 0.05, **P ≤ 0.01, ***P ≤ 0.001, ****P ≤ 0.0001. The schematic was created in BioRender.com.

Healthy children (*n* = 110) aged 5–12 years were recruited between 20 December 2021 and 8 March 2022 and followed up for 1 year. They had neither clinical history nor serological or T cell evidence of previous SARS-CoV-2 infection and received two doses of 10 μg BNT162b2 (Fig. 1a). The vaccination period coincided with the emergence of the Omicron variant in Singapore, at a time when the majority of the adult population had already been fully vaccinated. Our pediatric cohort (demographic profile shown in Extended Data Table 1) thus provided a unique opportunity to glimpse unreported features of vaccine-induced and hybrid adaptive immunity against symptomatic Omicron infection.

### Immunogenicity of mRNA SARS-CoV-2 vaccines in children

**Extended Data Fig. 1:**
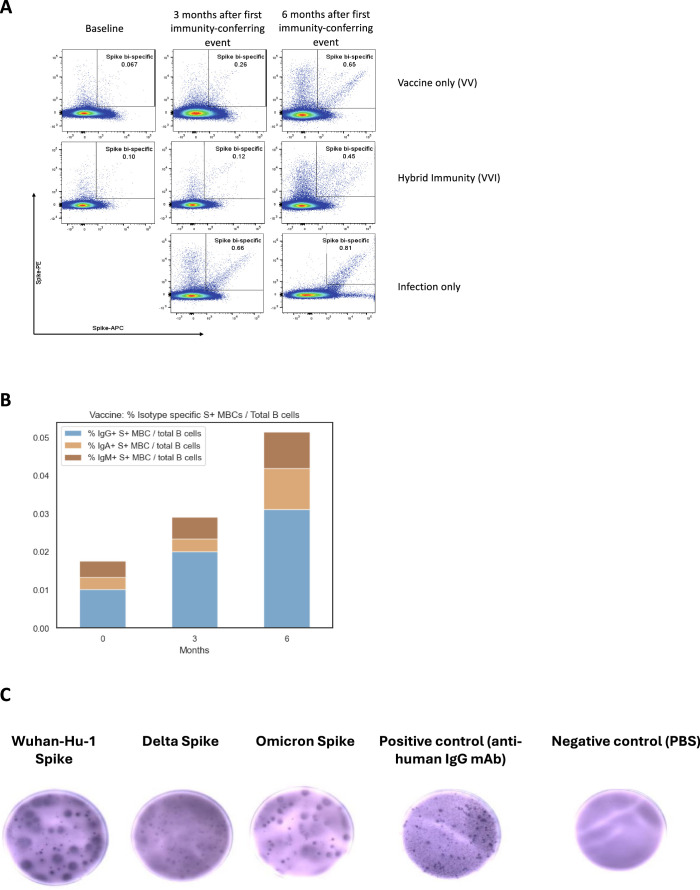
Isotypes and binding activity of Spike-specific memory B cells (S + MBC) in children with immunity against SARS-CoV-2. **A**. Representative flow cytometric staining plots for vaccine-only (VV), hybrid (VVI), and infection-only immunity (n = 1 each). **B**. S + MBC isotypes in children with vaccine-only immunity (n = 34), at pre-vaccination baseline, and 3- and 6 months post vaccination. **C**. Binding activity against Spike proteins from three SARS-CoV-2 variants in B cell ELISPOT. Spike-specific memory B cells (S+ MBCs) were extracted from a pediatric subject 3 months after 2 doses of mRNA BNT162b2, and then cultured and differentiated ex vivo into antibody secreting cells.

**Extended Data Fig. 2:**
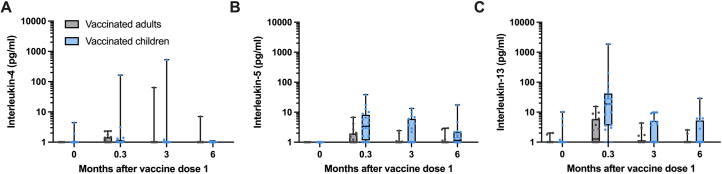
Children vaccinated with 2 doses of mRNA SARS-CoV-2 vaccine have low levels of Th2 cytokines (n = 34). **A, B, C**. Spike-reactive Th2 responses, measured by interleukin-4, interleukin-5 and interleukin-13 for children with vaccine-only immunity, and adults. For box-whisker graphs, upper and lower boundaries of boxes indicate upper and lower quartile respectively, line indicates median, and whiskers represent the range.

We first examined whether the lower pediatric dose of BNT162b2 generated adaptive immune responses comparable to those in healthy adults who received the full dose of 30 μg BNT162b2 in a parallel cohort^7^. Peripheral venous blood was sampled at the pre-vaccination baseline and 10 days, 3 months and 6 months after the first dose (Fig. 1a) for antibody, MBC, plasmablast and T cell measurements. Thirty-four children were included in the analysis as they had neither antigen rapid test-positive (ART^+^) symptomatic SARS-CoV-2 infection nor serological or T cell evidence of SARS-CoV-2 infection throughout the 6 months after vaccination (Fig. 1a). These children developed comparable levels of anti-S IgG antibodies at all time points as adults until 6 months after vaccination, whereupon the IgG levels were significantly higher in children than adults (Fig. 1b). Antibodies that neutralized 50% of S protein (Wuhan-Hu-1 only) receptor-binding domain (RBD) binding to angiotensin-converting enzyme 2 (ACE2), as measured using surrogate virus neutralization assay (sVNT_50_) were not detectable in either group until after the second dose of vaccination (Fig. 1c). S-specific (S^+^) MBCs increased over 3–6 months after vaccination and were marginally lower in children compared to adults at 6 months (Fig. 1d and Extended Data Fig. 1a). S^+^ MBCs were mainly IgG positive (Extended Data Fig. 1b) and bound to SARS-CoV-2 variants (Extended Data Fig. 1c). S^+^ plasmablasts were highest at day 10 after dose one, then decreased in number in circulation over 3–6 months after vaccination, likely reflecting their entry into bone-marrow niches to form long-lived plasma cells; the plasmablast response at 6 months was, like S^+^ MBCs, higher in adults (Fig. 1e). S-reactive T cell response, as measured by interferon (IFN)γ using a previously reported cytokine release assay^8^, was higher in children than adults at 3 and 6 months after vaccination (Fig. 1f), whereas interleukin (IL)-2 levels were similar across all time points in adults and children (Fig. 1g). Release of the type 2 helper T (T_H_2) cytokines IL-4, IL-5 and IL-13 was low (Extended Data Fig. 2a–c). No correlation was found between age, sex or weight of the children with any of the above adaptive immune parameters (Supplementary Table 1).

**Fig. 2:**
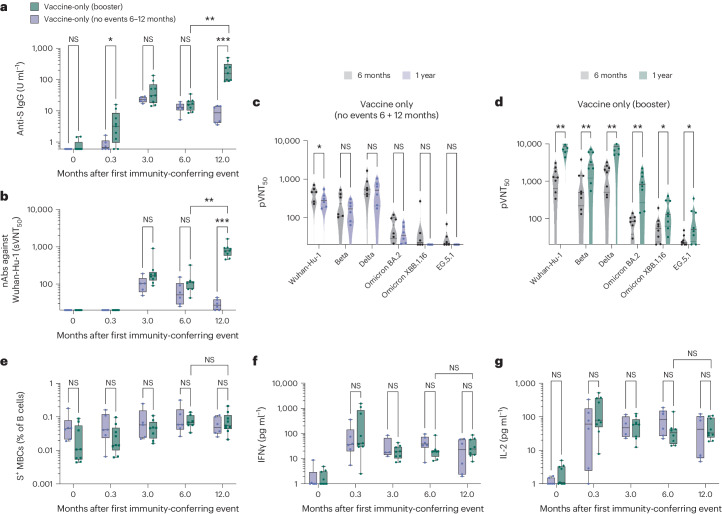
A booster dose increased antibody titers but had no effect on memory B cell and T cell responses for SARS-CoV-2-naive children. *n* = 6 vaccine-only children had no events between 6 and 12 months, whereas *n* = 9 children received a booster between 6 and 12 months. **a**,**b**, Anti-S IgG (**a**) and sVNT_50_ (**b**) titers at pre-vaccination baseline and day 10, 3 months and 6 months after vaccine dose one for SARS-CoV-2-naive children who received two or three doses of BNT162b2 over 1 year. **c**,**d**, Antibody titers that neutralized 50% of SARS-CoV-2 variants of concern (VOCs), quantified using pVNT_50_ in children without (**c**) and with (**d**) booster vaccination. **e**, Percentage of S^+^ MBCs out of total B cells in children with and without booster. **f**,**g**, S-reactive T cell responses measured by post-stimulation IFNγ (**f**) and IL-2 (**g**) levels in children with and without booster. For box-and-whisker graphs, the top and bottom boundaries of boxes indicate the upper and lower quartiles, respectively, the line indicates the median and whiskers represent the range. For all panels, a two-tailed Mann–Whitney *U*-test was used for comparisons between children who received two doses and those who received three doses (with booster) and a Wilcoxon rank test was used to compare parameters of the same individuals at 6 and 12 months. NS, not significant, *P ≤ 0.05, **P ≤ 0.01, ***P ≤ 0.001, ****P ≤ 0.0001.

**Extended Data Fig. 3:**
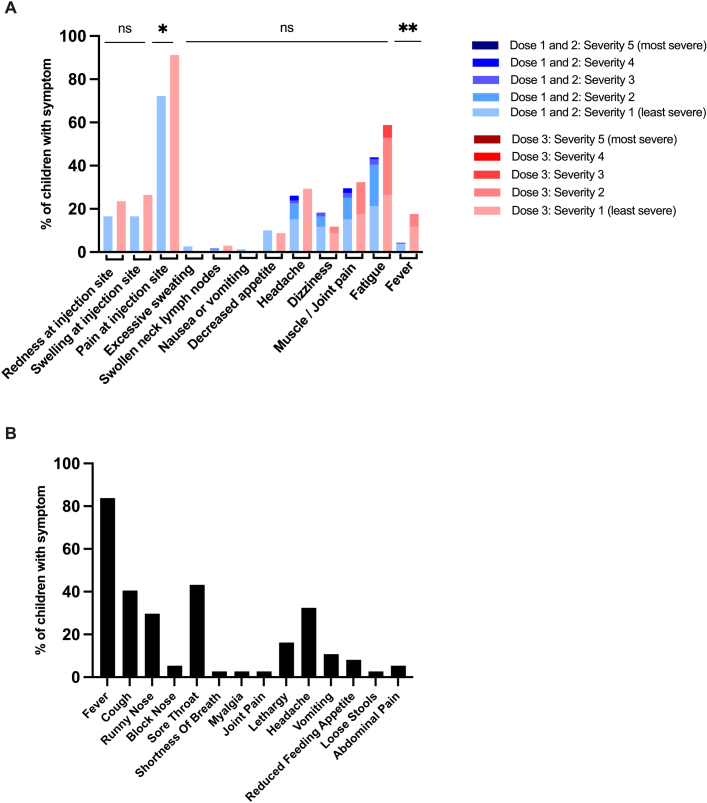
Vaccine-associated local and systemic adverse events and symptoms of SARS-CoV-2 infection reported by MARVELS children. **A**. Percentages and severity of parent and / or subject – reported local and systemic symptoms for 10 days after vaccination. Blue = adverse events after doses 1 and 2, with color gradient representing spectrum of severity; Red = adverse events after booster (dose 3), with color gradient representing spectrum of severity. **B**. Clinical features of symptomatic SARS-CoV-2 infections for all episodes of symptomatic SARS-CoV-2 infection among vaccinated children. Two-tailed Fisher’s exact test was used for comparison between groups; ns: not significant, *: p ≤ 0.05, **: p ≤ 0.01, ***: p ≤ 0.001, ****: p ≤ 0.0001. For pain at injection site, p-value = 0.0189. For fever, p-value = 0.0091.

Next, to determine the longevity of vaccine-induced adaptive immune responses, we identified 15 children who remained free of SARS-CoV-2 infection at the end of the 1-year follow up. Of these 15 children, 9 received a third dose of BNT162b2 (booster) and 6 did not (Fig. 1a). The booster dose increased anti-S IgG (Fig. 2a) and sVNT_50_ titers (Fig. 2b). A pseudotype virus neutralization test (pVNT) revealed increased titers of antibodies that neutralized 50% of Wuhan-Hu-1, as well as Beta, Delta and Omicron subvariant inoculum (pVNT_50_) (Fig. 2c,d). The booster had no effect on MBC (Fig. 2e) and T cell responses (Fig. 2f,g); however, the booster dose elicited more adverse events than doses one and two (Extended Data Fig. 3a). Taken collectively, these findings show that despite the lower dose of 10 μg, the humoral and cellular immune responses in children were comparable to those in adults, and that T and B cell memory responses remain stable over time with no benefit from a booster.

### Hybrid immunity in children

**Fig. 3:**
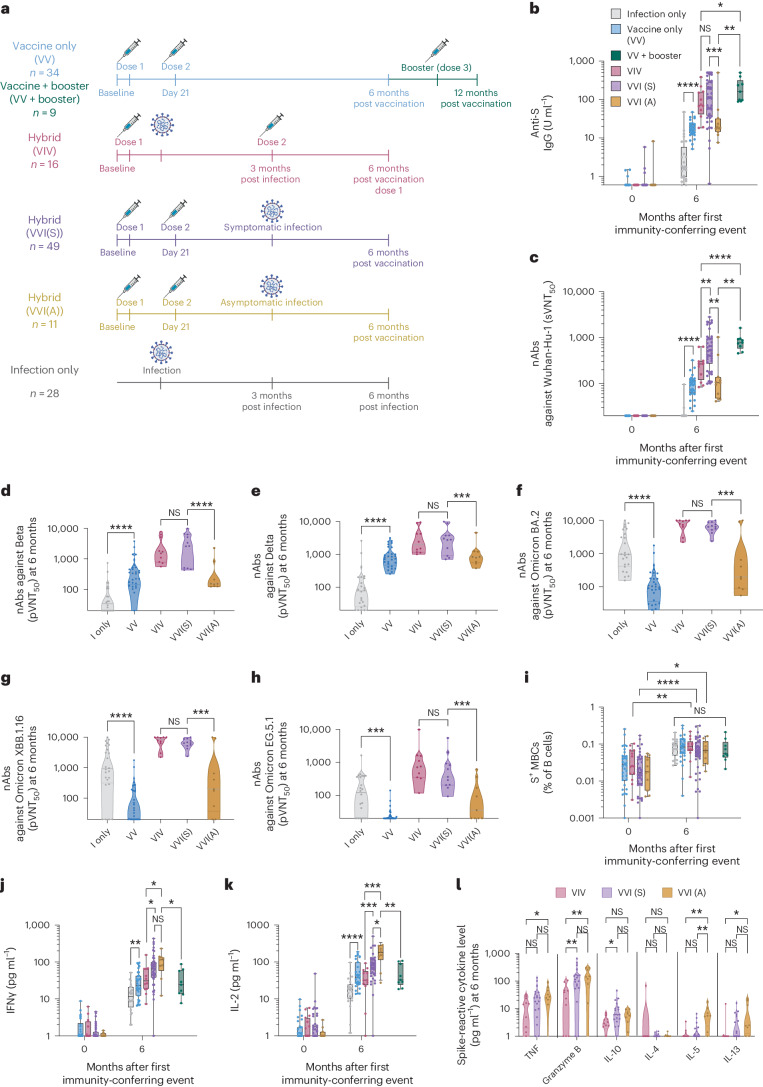
Superiority of the adaptive immune parameters in children who acquired hybrid immunity within 6 months post-vaccination compared to vaccination or infection alone. **a**, Schematic representation. A total of 110 children received two doses of 10 μg monovalent BNT162b2 at day 0 and day 21, and 28 unvaccinated children were naturally infected during a period when Omicron subvariants were dominant in the community. Of the vaccinated children, 34 had immunity conferred only by two doses of vaccine by 6 months (VV). Sixteen children had one dose of vaccine, followed by natural infection, followed by dose two 3 months later, which was given after the 3-month venepuncture (VIV). Sixty children had two doses of vaccine followed by a natural infection either before or after the 3-month venepuncture (VVI). S, symptomatic (*n* = 49); A, asymptomatic (*n* = 11). Twenty-eight unvaccinated children, who naturally acquired symptomatic SARS-CoV-2 infection, were used for comparison. The immunological parameters at 12 months of the VV children who received a booster but did not acquire infection between 6 and 12 months are displayed as VV + booster (*n* = 9). **b**, Anti-S IgG titers at pre-vaccination baseline and 6 months after the first immunity-conferring event. **c**–**h**, sVNT_50_ titers against Wuhan-Hu-1 (**c**) and pVNT_50_ titers against SARS-CoV-2 Beta (**d**), Delta (**e**), Omicron BA.2 (**f**), Omicron XBB.1.16 (**g**) and Omicron EG.5.1 (**h**) variants, at month 6. **i**, Percentage of S^+^ MBCs out of total B cells. **j**,**k**, S-reactive T cell responses measured by post-stimulation IFNγ (**j**) and IL-2 (**k**) levels. **l**, Concentration of indicated cytokines secreted in the 8-cytokine release assay for S-reactive T cell responses from children VIV, VVI(S) and VVI(A). For box-and-whisker graphs, the top and bottom boundaries of the boxes indicate upper and lower quartiles, respectively, the line indicates the median and whiskers represent the range. A two-tailed Mann–Whitney *U*-test was used to compare two groups. A Kruskal–Wallis H test was used to compare all groups for S^+^ MBC. NS, not significant, *P ≤ 0.05, **P ≤ 0.01, ***P ≤ 0.001, ****P ≤ 0.0001. The schematic was created in BioRender.com.

**Extended Data Fig. 4:**
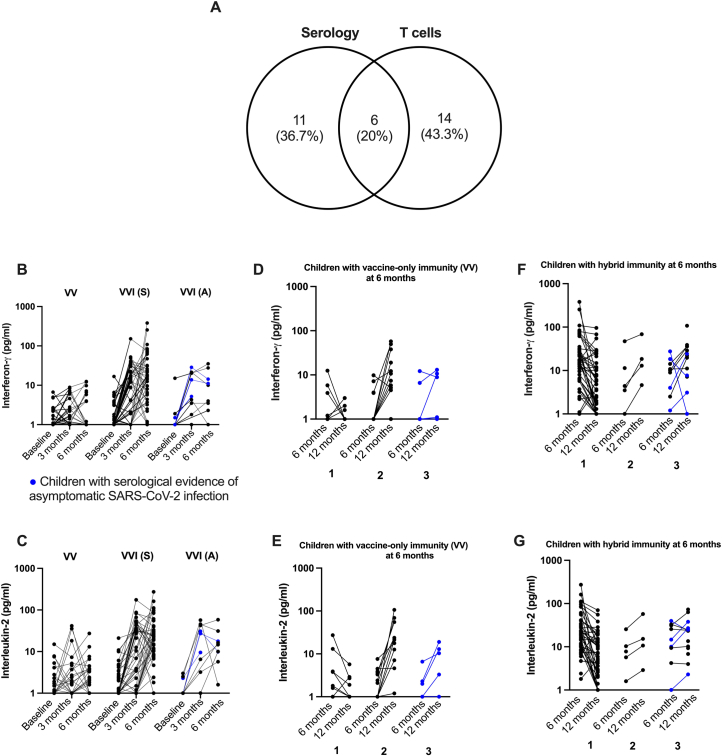
Nucleocapsid (N)-specific antibodies and T cells in children with asymptomatic SARS-CoV-2 infections. **A**. Venn diagram showing all episodes of asymptomatic COVID-19 identified in this study (n = 30), separated into whether they were identified via seropositivity, T cell reactivity, or both against SARS-CoV-2 N protein. **B, C**. N-reactive T cell responses measured by post-stimulation interferon-*γ* and interleukin-2 levels, at pre-vaccination baseline, 3- and 6 months post vaccination, from vaccinated (two doses) children who remained uninfected (VV, n = 34), with symptomatic infection (VVI(S), n = 49) and asymptomatic infection (VVI(A), n = 11). **D**. N-reactive T cell responses measured by post-stimulation interferon-*γ* and **E**. interleukin-2 levels, at 6- and 12 months post vaccination, for VV children at 6 months. **F**. N-reactive T cell responses measured by post-stimulation interferon-*γ* and **G**. interleukin-2 levels, at 6- and 12 months post vaccination, for children with hybrid immunity at 6 months. In **D-G**, children were grouped into the following categories: 1) Those who did not develop SARS-CoV-2 infection between 6 and 12 months, 2) those who developed symptomatic SARS-CoV-2 re-infection (S), or 3) those who had asymptomatic re-infection. In all graphs, subjects with serological evidence of asymptomatic SARS-CoV-2 are colored in blue.

As transmission of the Omicron variant (predominantly BA.2) in Singapore coincided with this study, we took advantage of this epidemiological opportunity to define hybrid immunity produced from different combinations of vaccination and infection (Fig. 3a). The observation period was within 6 months from the first BNT162b2 dose. A total of 76 vaccinated children were infected either symptomatically or asymptomatically as confirmed using serology and T cell responses. Among these, 16 children had symptomatic SARS-CoV-2 infection after the first vaccine dose; these children received their second dose 3 months after infection and were denoted as VIV (Fig. 3a). The remaining 60 children acquired SARS-CoV-2 infection after having completed two vaccine doses 21 days apart (denoted as VVI); of these, 49 (81.7%) were symptomatic (VVI(S)) and 11 (19.3%) were asymptomatic (VVI(A)) (Fig. 3a). All episodes of symptomatic infection among vaccinated children were mild (Extended Data Fig. 3b). Of asymptomatic infection episodes identified, 36.7% only had serological evidence of infection, whereas 43.3% only had nucleocapsid (N)-specific T cell responses (Extended Data Fig. 4a), confirming the previously reported importance of using both criteria to identify asymptomatic infections^9–11^. The trend of N-reactive T cell responses of asymptomatically infected children was similar to those symptomatically infected (Extended Data Fig. 4b–g).

**Extended Data Fig. 5:**
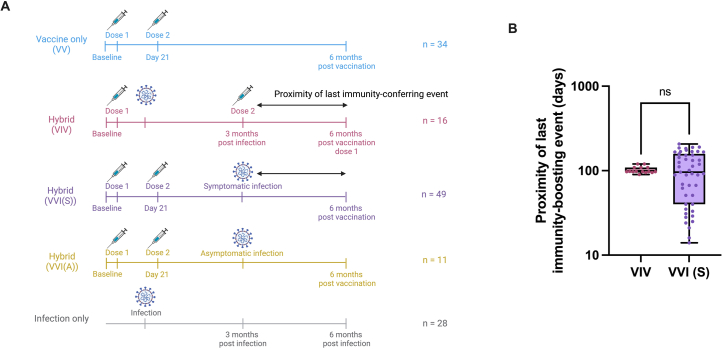
The proximity of last immunity-boosting event is similar for children who had symptomatic SARS-CoV-2 infection between doses 1 and 2 (VIV), and children who had symptomatic SARS-CoV-2 infection after 2 doses (VVI(S)). **A**. Schematic representation of the different last immunity-boosting event, for VIV and VVI(S) children. Created with BioRender.com. **B**. Comparison of proximity of immunity-boosting event for VIV and VVI(S) children. For box-whisker graphs, upper and lower boundaries of boxes indicate upper and lower quartile respectively, line indicates median, and whiskers represent the range. Two-tailed Mann–Whitney U test was used for comparison between groups; ns: not significant, *: p ≤ 0.05, **: p ≤ 0.01, ***: p ≤ 0.001, ****: p ≤ 0.0001.

To determine whether the infection before or after completion of the two doses, as well as symptom manifestation, affected the level of hybrid immunity, we compared the month-6 immunological parameters found in three groups with hybrid immunity to those in the 34 vaccinated but uninfected children, denoted as VV. The month-12 immunological parameters of the nine VV children who remained uninfected throughout the study but received a booster between months 6 and 12, denoted as ‘VV + booster’, were also included for direct comparison of hybrid immunity to vaccine-only immunity after a total of three doses (Figs. 1a and 3a). An additional 28 unvaccinated but infected children were also included as controls (Fig. 3a). Except for S^+^ MBCs, wild-type infection produced adaptive immune responses that were universally lower than those elicited by vaccination, whereas hybrid immunity produced the highest adaptive immune responses (Fig. 3b–k). This is similar to what has previously been reported in adults^1,12–14^. Among children with hybrid immunity, VVI(S) had the highest sVNT_50_ titers (Fig. 3c) at 6 months, higher than VIV children even though there was no difference in the proximity of last immunity-boosting event to the month-6 sampling time point (Extended Data Fig. 5). We found no difference in pVNT_50_ titers against the SARS-CoV-2 variants between VIV and VVI(S) (Fig. 3d–h), suggesting the possibility that the sequence of vaccination and infection only affects the anamnestic B cell response but not the development of new neutralizing antibodies against SARS-CoV-2 variants. VVI(A) produced the lowest anti-S IgG (Fig. 3b) and sVNT_50_ titers (Fig. 3c) among children with at least three episodes of antigenic exposure (VIV, VVI(S) and VV + booster). Similarly, pVNT_50_ titers against the different SARS-CoV-2 variants were universally lower in VVI(A) than in either VIV or VVI(S) (Fig. 3d–h). MBC responses were, however, comparable between all groups of children with hybrid immunity (Fig. 3i).

**Extended Data Fig. 6:**
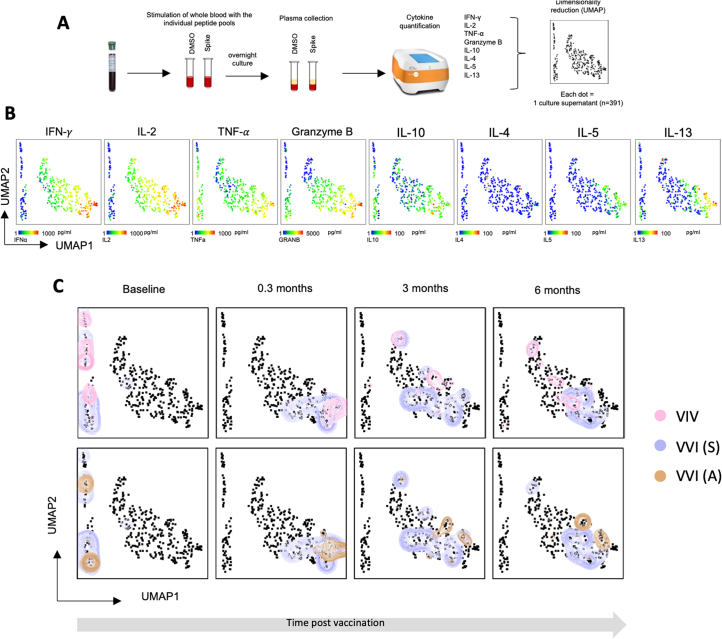
Eight-cytokines measured in the cytokine release assay for Spike-reactive T cell responses among children with hybrid immunity. **A**. Schematic of cytokine release assay and analytical methods (after DMSO control subtraction) using unsupervised clustering algorithm (UMAP). The cytokines quantified were interferon-*γ* (IFN-*γ*), interleukin-2 (IL-2), tumor necrosis factor-α (TNF-α), Granzyme-B, interleukin-10 (IL-10), interleukin-4 (IL-4), interleukin-5 (IL-5), and interleukin-13 (IL-13). **B**. UMAP plots generated with all analyzed samples (n = 391, consisting of adults, infected children and vaccinated children at all time points) with levels of secreted cytokines shown in heatmaps. **C**. Concatenated cytokine secretion profiles of S peptide pool-stimulated whole blood from children with VIV, VVI(S) and VVI(A). Cytokine secretion profiles were overlaid on the global UMAP plot of all analyzed samples (black dots; each dot corresponds to one culture supernatant).

Besides the B cell responses, the 8-cytokine release assay showed superiority of T cell responses against epitopes on the S protein (Extended Data Fig. 6a,b) in VVI(S) and VVI(A) compared to VIV (Extended Data Fig. 6c). Both VVI(S) and VVI(A) children had higher secretion of IFNγ, IL-2 and granzyme-B in the cytokine release assay than VIV children, suggesting a strong and polyfunctional T cell response (Fig. 3j–l and Extended Data Fig. 6c). Remarkably, VVI(A) children had the highest T_H_1 response, as measured by IL-2 (Fig. 3k).

Collectively, our findings suggest that hybrid immunity is best attained when infection occurs after completion of the two-dose primary vaccination. Symptomatic SARS-CoV-2 infection produced higher nAbs than asymptomatic infection, although both groups showed comparable T cell responses.

### Booster vaccination in children with hybrid immunity

**Fig. 4:**
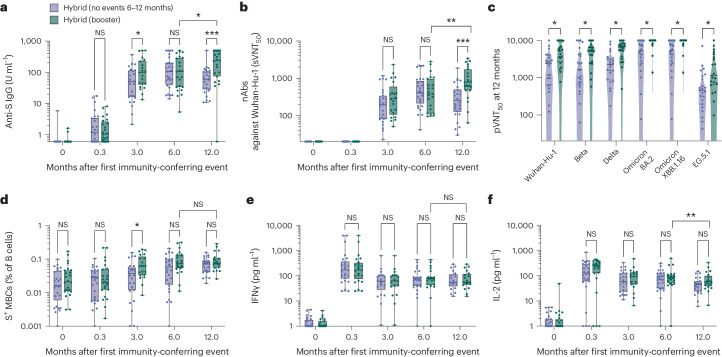
A booster dose had minimal effects on adaptive immune responses in children with hybrid immunity. *n* = 30 children with hybrid immunity had no events between 6 and 12 months, whereas *n* = 25 children received a booster between 6 and 12 months. **a**,**b**, Anti-S IgG (**a**) and sVNT_50_ titers (**b**) at pre-vaccination baseline and day 10, 3 months and 6 months after vaccine dose one, for children who developed hybrid immunity by month 6 and were not re-infected thereafter, with or without a booster dose. **c**, PVNT_50_ titers against SARS-CoV-2 variants at 12 months from start of vaccination. **d**, Percentage of S^+^ MBCs out of total B cells. **e**,**f**, Levels of S-reactive T cell responses measured by post-stimulation IFNγ (**e**) and IL-2 (**f**). For box-and-whisker graphs, the top and bottom boundaries of the boxes indicate the upper and lower quartiles, respectively, the line indicates the median and whiskers represent the range. For all panels, a two-tailed Mann–Whitney *U*-test was used for comparisons between children who received two doses and those who received three doses (with booster) and a Wilcoxon rank test was used to compare parameters of the same individuals at 6 and 12 months. NS, not significant, *P ≤ 0.05, **P ≤ 0.01, ***P < 0.001, ****P < 0.0001.

Of the 76 children who acquired hybrid immunity from either VIV, VVI(S) and VVI(A) groups by 6 months, 55 were not re-infected between 6 and 12 months; of these, 25 received a booster and 30 did not (Fig. 1a). At 12 months, the anti-S IgG and sVNT_50_ titers were higher in the children with booster vaccination compared to those without booster vaccination (Fig. 4a,b); however, the level of increase in these titers was small compared to the change between first and second doses of vaccination. Similarly, the booster dose increased pVNT_50_ titers against SARS-CoV-2 variants although the titers in children without boosters were already high (Fig. 4c). There was no difference in S^+^ MBC (Fig. 4d) and T cell responses as measured by IFNγ and IL-2 (Fig. 4e,f). Collectively, we found little to no immunogenic benefit, based on the responses we measured, in booster vaccination in children with hybrid immunity.

### Immune correlates of protection

Finally, we took advantage of our longitudinal blood sampling to determine the adaptive immunological parameters that correlated with protection against symptomatic SARS-CoV-2 infection. For this analysis, we divided our observation into two periods: between 3 and 6 months, and between 6 and 12 months after vaccination, which represented a population with mostly pre-hybrid and post-hybrid immunity, respectively. The rationale for such analyses is that, unlike adults who would mostly have been vaccinated and infected with SARS-CoV-2, future newly vaccinated children may yet encounter their first infection. Identifying the pre-hybrid immune correlates of protection, especially in the presence of an antigenic mismatch between the vaccine and SARS-CoV-2 variant, could thus be informative on how well vaccine-induced immunity protects against symptomatic SARS-CoV-2 infection in the future.

**Fig. 5:**
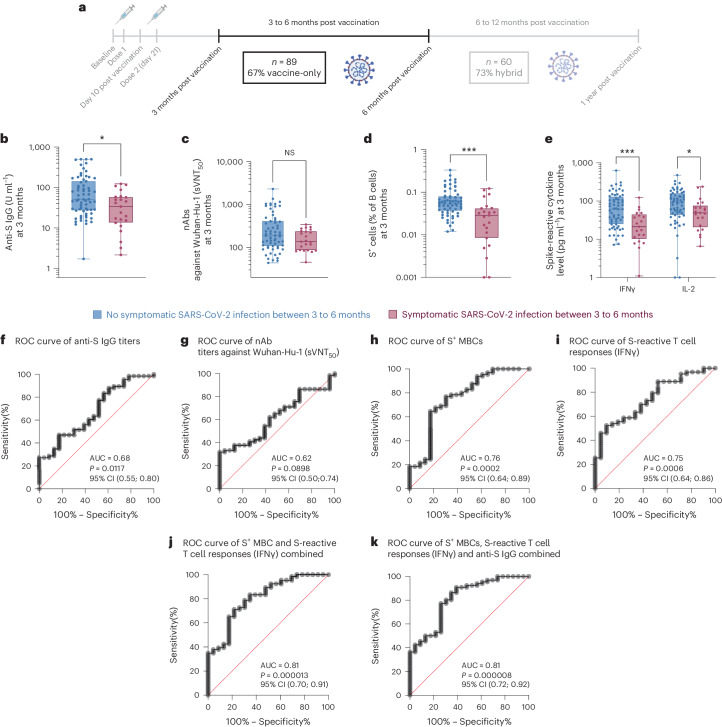
Anti-S IgG S^+^ MBCs and T cell responses are correlates of protection against symptomatic SARS-CoV-2 infection before hybrid immunity. *n* = 23 children had symptomatic SARS-CoV-2 infection between months 3 and 6, whereas *n* = 66 did not. **a**, Timeline showing time intervals during which vaccine-elicited immune parameters were analyzed against symptomatic SARS-CoV-2 infection. **b**–**e**, 3-month anti-S IgG titers (**b**), sVNT_50_ titers against Wuhan-Hu-1 (**c**), percentage of S^+^ MBCs out of total B cells (**d**) and S-reactive T cell responses (**e**) measured by post-stimulation IFNγ and IL-2 levels, in children who did or did not develop symptomatic SARS-CoV-2 infection between 3 and 6 months. For box-and-whisker graphs, the top and bottom boundaries of the boxes indicate upper and lower quartiles, respectively, the line indicates the median and whiskers represent the range. A two-tailed Mann–Whitney *U*-test was used to compare groups. NS, not significant, *P < 0.05, **P < 0.01, ***P < 0.001, ****P < 0.0001. **f**–**k**, ROC curve for anti-S IgG titers (**f**), sVNT_50_ titers (**g**), S^+^ MBCs (**h**), IFNγ (**i**), S^+^ MBCs and IFNγ combined (**j**) and S^+^ MBCs, IFNγ and anti-S IgG combined (**k**), with all parameters measured at 3 months from the start of vaccination. The ROC curve analysis was performed using the Wilson/Brown test. The timeline was created in BioRender.com.

**Extended Data Fig. 7:**
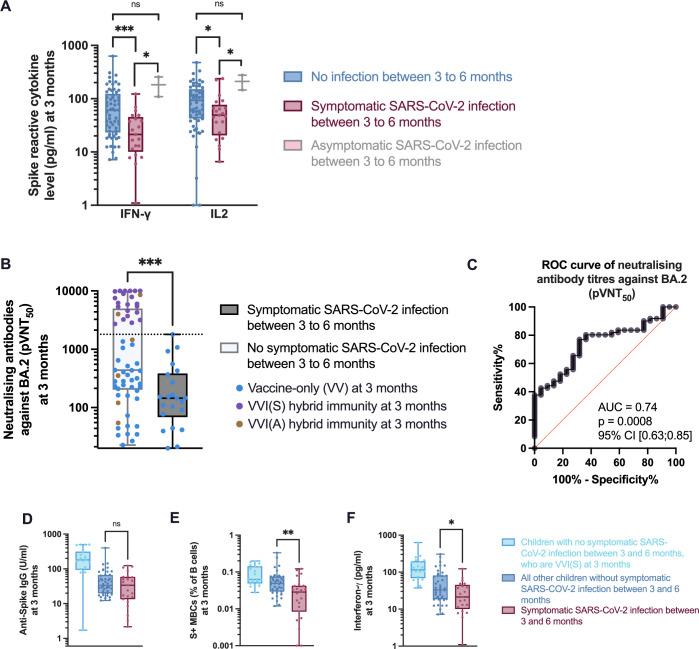
Immune correlates of protection against symptomatic SARS-CoV-2 infection between 3- and 6 months from start of vaccination. N = 23 children had symptomatic SARS-CoV-2 infection between months 3 and 6, while n = 66 did not; of these, n = 3 had asymptomatic SARS-CoV-2 infection. **A**. 3-month Spike (S)-reactive T cell responses, quantified using interferon- *γ* (IFN-*γ*) and interleukin-2 (IL-2), for children with no infection, symptomatic and asymptomatic SARS-CoV-2 infection between 3 and 6 months. For comparison between no infection and symptomatic infection, p-value for IFN-*γ* = 0.000688 and for IL-2 = 0.025786. For comparison between symptomatic infection and asymptomatic infection, p-value for IFN-*γ* = 0.015810 and for IL-2 = 0.031621. **B**. 3-month Omicron BA.2 pVNT_50_ titers in children who did and did not develop symptomatic SARS-CoV-2 infection between 3 and 6 months. Color of dots indicate the type of pre-existing immunity they had at month 3 (p-value = 0.000589). **C**. Receiver operator characteristics (ROC) curve for BA.2 measured at 3 months from start of vaccination. **D**. pVNT50 titers Anti-S IgG titers, **E**. Percentage of S+ MBCs out of total B cells (p-value 0.004599), and **F**. S-reactive T cell responses measured by post-stimulation interferon-*γ* levels at month 3 post-vaccination (p-value 0.047200), for VVI(S) and non-VVI(S) children with and without symptomatic SARS-CoV-2 infection. For box-whisker graphs, upper and lower boundaries of boxes indicate upper and lower quartile respectively, line indicates median, and whiskers represent the range. Two-tailed Mann–Whitney U test was used to compare between groups. The ROC curve analysis was performed using the Wilson/Brown test. ns: not significant, *: p ≤ 0.05, **: p ≤ 0.01, ***: p ≤ 0.001, ****: p ≤ 0.0001.

In the first period, after excluding VIV children as they received their second dose within this period, data from 89 children were analyzed (Fig. 5a). The majority (60 of 89 = 67%) of the cohort had vaccine-only immunity 3 months after vaccination. Symptomatic SARS-CoV-2-infected children (*n* = 23) had lower anti-S IgG (Fig. 5b) at month 3, but not sVNT_50_ titers (Fig. 5c), than those without symptomatic SARS-CoV-2 infection (*n* = 66). Symptomatically infected children also showed lower S^+^ MBCs (Fig. 5d) and T cell responses at month 3 as demonstrated by lower secretion of IFNγ and IL-2 in the cytokine release assay (Fig. 5e) than those without symptomatic infection. Notably, children who showed serological and T cell evidence of SARS-CoV-2 infection, but did not report symptoms, had higher T cell responses than those with symptoms (Extended Data Fig. 7a).

While sVNT_50_ titers were not higher in those without than with symptomatic infection, a significant receiver operating characteristic (ROC) curve and high BA.2 pVNT_50_ titers were due to VVI(S) children (Extended Data Fig. 7b,c). Removal of children with VVI(S)-associated hybrid immunity from our analysis showed no difference in anti-S IgG titers between children with and without symptomatic infection (Extended Data Fig. 7d). The S^+^ MBC and S-reactive T cell responses, however, remained significantly higher in children without than with symptomatic infection (Extended Data Fig. 7e,f). Our findings thus suggest the potential of MBCs and T cells to protect against symptomatic infection in those without high pre-existing and variant-specific nAbs.

To identify the correlates of protection from symptomatic infection when most of the population have yet to develop hybrid immunity or variant-specific neutralizing antibodies, we plotted the ROC curve for the different immunological parameters measured at 3 months from the start of vaccination. The area under the ROC curves (AUC) of anti-S IgG titers (Fig. 5f) but not sVNT_50_ (Fig. 5g) was statistically significant. Likewise, S + MBC counts and T cell response, as reflected by IFNγ release levels at 3-month after the start of vaccination, were also predictive of protection against symptomatic infection (Fig. 5h,i). Combinations of S+ MBCs and T cell (IFNγ release) levels (Fig. 5j) and anti-S IgG titers (Fig. 5k) increased the ROC AUC values although the 95% confidence interval (CI) overlapped with those shown in Fig. 5h,i. Finally, multivariate linear regression revealed T cell responses (IFNγ release) to be the most important predictor for protection against symptomatic SARS-CoV-2 infection (Extended Data Table 2).

**Fig. 6:**
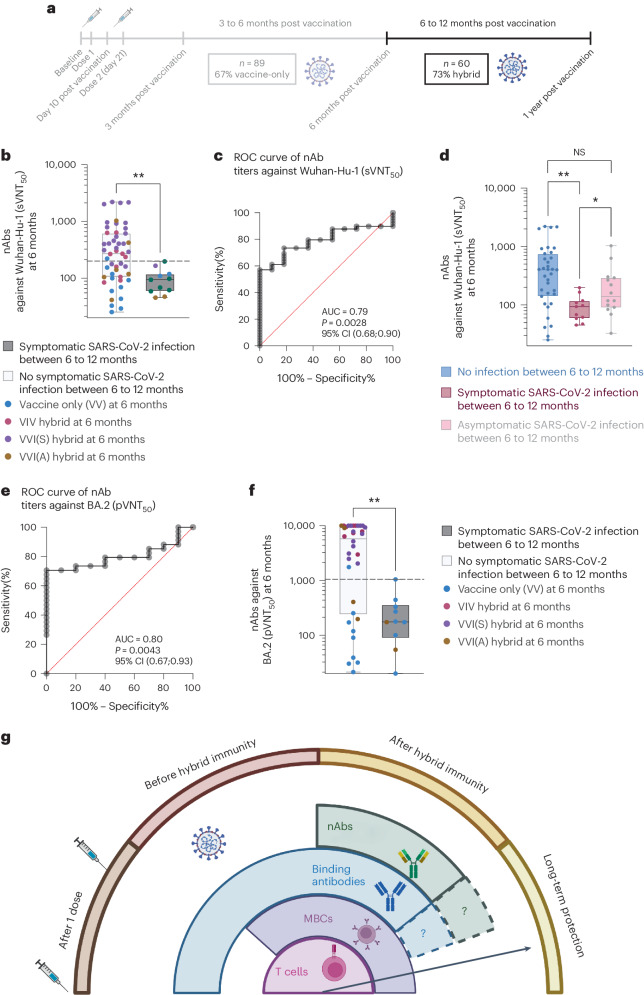
nAb titers correlate with protection against symptomatic SARS-CoV-2 infection when prevalence of hybrid immunity was high. *n* = 11 children had symptomatic SARS-CoV-2 infection between 6 and 12 months, whereas *n* = 49 children did not; of these, *n* = 14 had asymptomatic SARS-CoV-2 infection. **a**, Timeline showing time interval of analysis. **b**, 6-month sVNT_50_ titers against Wuhan-Hu-1 in children who did or did not develop symptomatic SARS-CoV-2 infection between 6 and 12 months. **c**, ROC for sVNT_50_ measured at 6 months from the start of vaccination. **d**, 6-month sVNT_50_ titers in children who had no infection, symptomatic and asymptomatic SARS-CoV-2 infection between 6 and 12 months. **e**, ROC for Omicron BA.2 pVNT_50_ measured at 6 months from the start of vaccination. **f**, 6-month Omicron BA.2 pVNT_50_ titers in children who did and did not develop symptomatic SARS-CoV-2 infection between 6 and 12 months, differentiated by the type of pre-existing immunity that they had at month 6. For box-and-whisker graphs, the top and bottom boundaries of boxes indicate the upper and lower quartiles, respectively, the line indicates the median and whiskers represent the range. A two-tailed Mann–Whitney *U*-test was used to compare groups. The ROC curve analysis was performed using the Wilson/Brown test. NS, not significant, *P < 0.05, **P < 0.01, ***P < 0.001, ****P < 0.0001. **g**, ‘Onion model’ of immune correlates of protection. The timeline and ‘onion model’ were created in BioRender.com.

**Extended Data Fig. 8:**
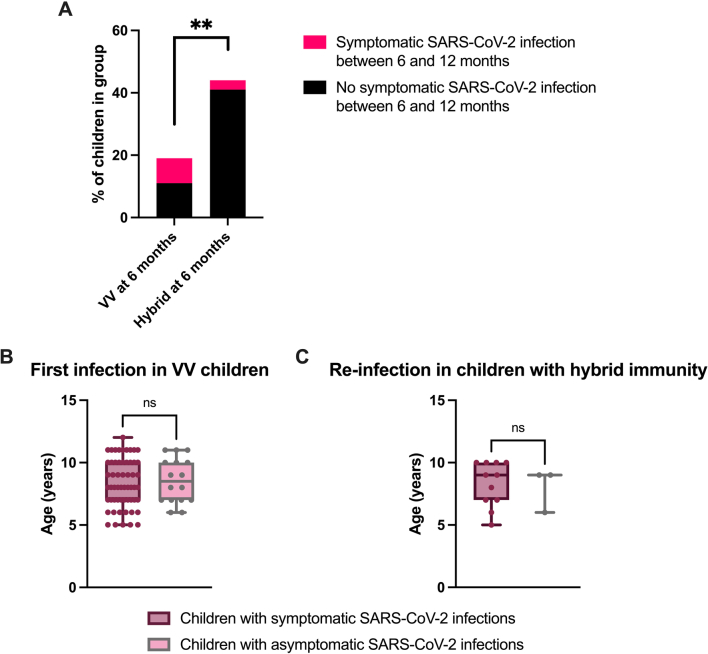
Protection from symptomatic SARS-CoV-2 infection was associated with hybrid immunity but not age. **A**. Superior protective capacity of hybrid immunity, demonstrated by the different in percentages of children who developed symptomatic SARS-CoV-2 infection between 6 and 12 months, among children with vaccine-only (VV) immunity at 6 months, compared to children with hybrid immunity at 6 months. Two-tailed Fisher’s exact test was used for comparison between groups. P-value = 0.0018. **B, C**. Symptomatic SARS-CoV-2 infection was not associated with age, as demonstrated by **B**. age distribution of all children getting their first infection after 2 doses of mRNA vaccination (n = 57 symptomatic, n = 16 asymptomatic), and **C**. age distribution of all children with hybrid immunity getting a re-infection (n = 3 symptomatic and n = 11 asymptomatic). For box-whisker graphs, upper and lower boundaries of boxes indicate upper and lower quartile respectively, line indicates median, and whiskers represent the range. Two-tailed Mann–Whitney U test was used to compare between different groups. ns: not significant, *: p ≤ 0.05, **: p ≤ 0.01, ***: p ≤ 0.001, ****: p ≤ 0.0001.

The protective effect of nAbs is evident only in the second period of observation, when the majority of children had already acquired hybrid immunity (Fig. 6a). After excluding children who received booster vaccination during this period, 60 children were available for analysis. Of these, 44 (73%) had hybrid immunity at the start of the period of observation. Of the 11 episodes of symptomatic SARS-CoV-2 infections, only 3 were re-infections among children with pre-existing hybrid immunity, whereas 8 episodes were first infections among VV children (relative risk 0.17, 95% CI 0.05–0.51) (Extended Data Fig. 8a). This finding was not confounded by the age of the children, which had no effect on symptomaticity of SARS-CoV-2 infections (Extended Data Fig. 8b,c). SVNT_50_ titers were significantly higher in children without than with symptomatic infection (Fig. 6b) and were predictive of protection against symptomatic infection (Fig. 6c). Likewise, sVNT_50_ titers were higher in infected children who remained asymptomatic than those who were symptomatic (Fig. 6d). BA.2 pVNT_50_ titers were also predictive of protection against symptomatic SARS-CoV-2 infection, but did not improve on predictive capacity (Fig. 6e). Notably, full protection against symptomatic BA.2 infection seems to require a relatively high pVNT_50_ titer of 1,000, which was achieved in all children with VVI(S) hybrid immunity but not the other combinations of vaccination and infection (Fig. 6f); even three doses of mRNA vaccination did not produce pVNT_50_ titers that approached this threshold (Fig. 2d). As nAbs blocked infection, the activity of the other components of adaptive immune responses in protecting against symptomatic infection would be masked and could not thus be compared meaningfully.

Taken collectively, the correlates of protection depended on whether adaptive immunity in a population was mostly produced through vaccination alone or through a combination of vaccination and infection. Our findings are summarized in Fig. 6g and discussed below.

## Discussion

The endemicity of COVID-19, the continual emergence of new variants of SARS-CoV-2 and introduction of new susceptible individuals with each birth cohort necessitate evidence to inform COVID-19 childhood vaccination policies. This study examined the different compartments of the adaptive immune response to mRNA vaccination that, along with the 1-year long observation period, provides hitherto missing insights into the humoral and cellular immune correlates of protection, both before and following hybrid immunity.

Indeed, the lack of evidence on long-term protection offered by vaccination has led to variability in pediatric clinical practice guidelines across the world. The US Centers for Disease Control and Prevention has recommended only one dose of mRNA vaccine as a primary vaccination of children aged 5 years and older, but also as a single booster dose for children who have received any number of doses of previous vaccines^15^. The UK Joint Committee on Vaccinations and Immunizations has supported primary vaccination of 5–11-year-olds with two doses of mRNA vaccine at least 12 weeks apart and without boosters for children who are not at risk for severe disease^16^. The European Medical Agency has approved mRNA vaccines to be used both as primary vaccination and boosters, but recommendations across European countries are heterogenous^17^. In Singapore, an additional booster vaccine is recommended a year after the primary two-dose vaccination series in children aged 5–17 years^18^. Our findings thus, fill a gap in knowledge necessary to inform and harmonize a pediatric vaccination strategy.

The variability in vaccination policies has also been accentuated by the almost singular focus on nAbs as the mediator of protection, at the exclusion of the other compartments of the adaptive immune system^3,19–24^. This has occurred despite clinical and epidemiological evidence of protection by cellular immunity against severe COVID-19 and even symptomatic SARS-CoV-2 infection^5,25–28^. Moreover, SARS-CoV-2 is constantly evolving to escape nAbs^29^. Consequently, until a pan-BetaCoV vaccine is developed, mismatch in the nAb epitopes on the vaccine and new SARS-CoV-2 variants is to be expected. Our data suggest that anti-S IgG, S^+^ MBCs and T cells can protect against symptomatic SARS-CoV-2 infection at a time when nAbs are insufficient to prevent infection. Critically, MBC and T cell levels remained stable over time, indicating that the levels attained after completion of the two doses of vaccination can inform individual protection against symptomatic SARS-CoV-2 infection. Furthermore, we found T cell levels to be the single most predictive factor. Notably, it is the anamnestic T cell response that is the earliest during a breakthrough infection^30^. Moreover, the nAbs produced after booster vaccination are well below those in children with VVI(S) hybrid immunity, which is the only group with antibody titers above the threshold associated with protection from symptomatic infection. Collectively, our findings question current pediatric vaccination guidelines that call for booster vaccination in children who have completed two-dose mRNA vaccination; the higher rates of adverse events from booster compared to primary vaccination in our cohort further cautions on the possibility of risks outweighing benefits.

In this pediatric cohort, we found that hybrid immunity produced the highest level of antibody and T cell responses against SARS-CoV-2 (refs. ^1,12–14,31–33^). Notably, we found that VVI was superior to VIV, especially in anamnestic B cell responses and T cell responses. This provides an immunological perspective underpinning recently reported epidemiological observations in 5–11-year-olds during the Omicron wave in Singapore, where VVI-type hybrid immunity had higher adjusted vaccine effectiveness than VIV-type hybrid immunity^34^. Primary vaccination of future birth cohorts should thus aim for timely completion of two doses within a 3–4-week interval to minimize SARS-CoV-2 infection occurring in between the vaccine schedule.

Differences in pre- and post-hybrid immune correlates of protection further suggests an ‘onion model’ of protection (Fig. 6g). When sufficiently high titers of variant-specific nAbs are present, the protection offered by virus neutralization masks the roles played by the other elements of the adaptive immune system^2,35^. It thus forms the outermost layer of protection; however, high titers of nAbs, including those specific to the variant of concern, may be needed, which seems attainable only under VVI(S) conditions. In those without symptomatic infection after vaccination or when nAbs wane to subprotective levels, the inner layers formed by MBCs and T cells can still be relied upon to protect against symptomatic and severe SARS-CoV-2 infection^24,28,36,37^. This protective capacity of cellular immunological memory has also been demonstrated in patients on B cell depletion therapy such as rituximab; despite the lack of antibody production, the T cell responses produced by mRNA vaccination^38^ reduced their risk of severe COVID-19 (ref. ^39^).

Although the correlates of protection were defined in children, we believe that it can also inform protection against symptomatic SARS-CoV-2 infection in healthy adults. This notion is supported by our finding of mostly comparable B and T cell response to vaccination between children and adults; where differences were found, they were marginal in extent; however, adults with chronic diseases with greater susceptibility to COVID-19 may require a higher threshold of antibodies, MBCs and T cells to be protected against both symptomatic infection and severe disease than what we report in this study. Longitudinal studies to define correlates of protection in these at-risk populations could prove helpful to shaping booster vaccination policies to keep severe COVID-19 at bay.

A limitation of our study is the lack of information about mucosal immunity, which is an important compartment of protective immunity for children with an infection history with SARS-CoV-2 (ref. ^12^); recurrent natural infections may conceivably boost mucosal immunity even if the effect on systemic immunity is smaller. Mucosal immune parameters could further refine the correlates of protection that collectively could guide both future vaccine development and public health policies in COVID-19 control. With limited blood volume that can be drawn from children, we have used the cytokine release assay to measure S-reactive T cells. Although this assay has shown strong correlation with ELISpot in quantifying T cell response, it is not possible to delineate which cell type (for example, CD4^+^ versus CD8^+^ T cells) secreted the cytokines measured; however, both CD4^+^ and CD8^+^ T cells are protective during SARS-CoV-2 infection^26,30^. Finally, our study was conducted in a small sample size of 110 children with few asymptomatic infections. As symptoms were self-reported, very mild infections may have also been classified as asymptomatic. Larger studies could reveal additional nuances on SARS-CoV-2 immunity that may have been missed in this study.

The mRNA SARS-CoV-2 vaccines elicited a durable and holistic immune response in children. Primary vaccination of future birth cohorts should consist of a two-dose regimen administered in a 3–4-week intervals, without booster vaccination, as evidenced by S^+^ MBCs and T cells being correlates of protection against symptomatic SARS-CoV-2 infection before acquisition of hybrid immunity.

## Methods

### Study design

We conducted a prospective cohort study, MARVELS, in healthy children aged 5–12 years. The study protocol was approved by the National Healthcare Group Domain Specific Review Board (DSRB) (2021/00945 and 2021/00984). As the study was originally designed to study immunogenicity of mRNA vaccines in children, we aimed to recruit 100 children based on the numbers of individuals in other published studies^40–42^ and taking into account participant attrition rate. As the Omicron variant emerged after study initiation, providing a fortuitous opportunity for us to identify immune correlates of protection, there was no a priori power calculation performed for the identification of correlates of protection.

Children were recruited from the general population, through advertisements around the National University Hospital, Singapore and in the community with written parental consent and written participant assent. Demographic characteristics, including sex, were parent- or self-reported and were not considered for enrollment. The decision to receive the vaccination was based on parental discretion and children were inoculated with two doses of BNT162b2 at days 0 and 21. The interval between the first and second dose was extended to 3 months if the child acquired natural SARS-CoV-2 infection after dose one. Among children who completed their primary two-dose vaccination regime, several received a booster dose at 5 months or later after dose two, also at parental discretion. Participants were enrolled between 20 December 2021 and 8 March 2022.

All children underwent venepuncture after enrollment and before the first dose of BNT162b2 vaccination. Thereafter, vaccinated children underwent venepuncture at 10 days, 3 months, 6 months and 1 year. All recruited and vaccinated children were followed for up to 1 year. Children were excluded from the analysis if they were found to be seropositive or had SARS-CoV-2 nucleocapsid (N)-reactive T cell responses at baseline before vaccination.

In addition, unvaccinated children who had acquired symptomatic SARS-CoV-2 infection diagnosed with a contemporaneously administered ART were also recruited from the general population. These children underwent venepuncture at 3 and 6 months from the day of illness onset. The study protocol was approved by the National Healthcare Group DSRB (2022/00316); written parental consent and written participant assent were also obtained.

Adult participants were healthy healthcare workers recruited from the Singapore Health Services institutions and received 30 μg of BNT162b2 under the same schedule as the children, as previously described^7^. Convenience sampling was used for adults, with 18 to 20 individuals per comparison. The study protocol involving adults was approved by the SingHealth Centralized Institutional Review Board (CIRB/F 2021/2024) and written informed consent was obtained from all participants. All individuals in the pediatric and adult cohorts were compensated according to DSRB and Centralized Institutional Review Board guidelines.

Clinical data were collected in REDCap v.14.2.2 and exported for analysis into Microsoft Excel. Python v.3.9.2 was used to combine immunological parameters and clinical data.

### Identification of ART^+^ symptomatic SARS-CoV-2 infections

Parents were trained to administer a SARS-CoV-2 ART testing at home if the child developed symptoms suggestive of COVID-19 anytime during the 1-year follow-up period, according to prevailing national guidelines for active surveillance and community treatment during the COVID-19 pandemic in Singapore. ART kits were certified by the Health Sciences Authority of Singapore and freely distributed by the Ministry of Health. ART^+^ symptomatic SARS-CoV-2 infections were reported to the study team within 72 h and a symptom diary was completed by the study team based on a phone interview conducted within 72 h.

### Description of side effects after vaccination

Parents completed a symptom diary after vaccination for 10 days after every dose of mRNA vaccine administered to the child, with options for solicited and unsolicited side effects to be reported.

### Anti-S IgG and anti-N antibodies

Anti-S IgG was quantified using ELISA, as previously described^43^. In brief, two high-binding 96-well ELISA microplates (Greiner) were coated with 1 μg ml^−1^ Wuhan-Hu-1 S hexapro protein diluted in PBS and incubated at room temperature (RT) for 45 min. Plates were washed with PBS-T (0.05% Tween-20) and incubated with blocking buffer (PBS with 3% BSA) at RT for 1 h^43^. Plasma was diluted 200× and 5,000× in blocking buffer, while a standard antibody (anti-SARS-CoV-2 S RBD Neutralizing Antibody, Acrobiosystems) was serially diluted 10× for the standard curve. Blocked plates were washed and then incubated with diluted plasma and antibody standard in duplicate at RT for 1 h. Plates were washed and then incubated with HRP-IgG secondary antibody (Life Technologies) at 10,000× dilution at RT for 1 h. Finally, plates were washed and then the detection reagent 3,30,5,50-tetramethylbenzidine (Thermo Fisher) was added. The reaction was quenched with 1 M sulfuric acid/phosphoric acid. The sample optical density (OD) was measured with a spectrophotometer at 450 nm and concentrations in U ml^−1^ were interpolated from the standard curve using GraphPad Prism v.10.0.2.

Anti-N antibodies were detected using Elecsys Anti-SARS-CoV-2 immunoassay (Roche) for qualitative detection of total antibodies against the N antigen, which uses a sandwich ELISA against recombinant N. The manufacturer’s instructions were followed.

### sVNT (Wuhan-Hu-1 only)

nAbs against Wuhan-Hu-1 S were measured according to the manufacturer’s instructions using the commercial sVNT cPASS (GenScript), which is based on an ELISA measuring the binding of RBD to human ACE2 (refs. ^42,44,45^). This assay probes for antibodies inhibiting recombinant SARS-CoV-2 S protein binding to the hACE2 receptor. Technical duplicates were used for this assay. The percentage inhibition of RBD–hACE2 binding was computed using the following equation: percentage inhibition = (1 − ((OD of serum + RBD)/(OD of negative control + RBD))) × 100. As described by the cPASS kit, a cutoff of 20% was used as the lower limit of positivity. Samples were serially diluted until % inhibition was below 50% and the half-maximal inhibitory concentration was interpolated using GraphPad Prism v.10.0.2.

### pVNT (for Wuhan-Hu-1 and SARS-CoV-2 variants)

nAbs against VOCs were measured with a pVNT, as previously described^45,46^. Human lung carcinoma epithelial (A549, ATCC CRM CCL-185) cells were grown and maintained in RPMI-1640 supplemented with 10% FBS. The human *ACE2* gene in a pFUGW vector was introduced into A549 cells by lentivirus transduction and maintained in RPMI-1640 supplemented with 10% FBS and 15 µg ml^−1^ of blasticidin. Human embryo kidney (HEK293T; ATCC CRL-3216) cells were grown and maintained in DMEM supplemented with 10% FBS. SARS-CoV-2 parental (Wuhan-Hu-1), Beta, Delta, Omicron BA.2, Omicron XBB.1.16 (E180V, T478R) and EG.5.1 (F456L and Q52H) full-length spike pseudotyped viruses were produced by transfecting 20 µg of pCAGGS spike plasmid into 5 million HEK293T cells using FuGENE 6 (Promega). At 24 h after transfection, the transfected cells were infected with VSV∆G luc seed virus at a multiplicity of infection of 5 for 2 h. After two PBS washes, infected cells were replenished with DMEM 10% FBS supplemented with 1:5,000 diluted anti-VSV-G monoclonal antibody (clone 8GF11, Kerafast). Upon 80% cytopathic effect, pseudotyped viruses were collected by centrifugation at 2,000*g* for 5 min. Pseudoviruses (~3 million relative light units) were pre-incubated with fourfold serial diluted test serum in a final volume of 50 μl for 1 h at 37 °C, followed by infection of A549-ACE2 cells. At 20–24 h after infection, an equal volume of ONE-Glo luciferase substrate (Promega) was added and the luminescence signal was measured using the citation 5 microplate reader (BioTek) with Gen5 software v.3.10. The 50% neutralizing titer (NT_50_) was interpolated using GraphPad Prism v.10.0.2.

### Cytokine release assay for S- and N-reactive T cell responses

T cell responses were quantified with a cytokine release assay, a validated method of quantifying T cell responses that has good correlation to T cell ELISPOT^8^. Fresh peripheral blood was stimulated with 55 overlapping 15-mer peptide pools covering the immunogenic regions of the Wuhan-Hu-1 SARS-CoV-2 S protein (representing 40.5% of the whole S protein) (GenScript) before and after vaccination. A similar overlapping peptide pool of SARS-CoV-2 N protein (spanning the entire N protein) was also used to stimulate peripheral blood to test for previous SARS-CoV-2 infection. Freshly drawn whole blood was mixed with RPMI and stimulated with the indicated N or S peptide pool at 2 μg ml^−1^ or with 1.25% dimethylsulfoxide as a control. After 16 h of incubation, the supernatant (plasma) was collected and stored at −80 °C until analysis. Cytokine concentrations in the plasma were quantified using an Ella machine (ProteinSimple) with microfluidic multiplex cartridges that measured T_H_1-specific cytokines IFNγ and IL-2 for both adults and children. In addition, T_H_2-specific cytokines IL-4, IL-5 and IL-13 and other cytokines (TNF, granzyme-B and IL-10) were quantified for children, according to the manufacturer’s instructions (ProteinSimple). The levels of cytokines present in the plasma of dimethylsulfoxide controls were subtracted from the corresponding N- or S-stimulated samples. Technical duplicates were used for this assay.

Subsequently, concentrations of each cytokine in all culture supernatants were transformed using the logical transformation function, and Uniform Manifold Approximation and Projection (UMAP) was run using a 15 nearest neighbors, min_dist of 0.2 and Euclidean distance. The results obtained from the UMAP analysis were incorporated as additional parameters and converted to FCS files, which were then loaded into FlowJo to generate heatmaps of cytokine secretion on the reduced dimensions^9,10^.

### Isolation of PBMCs

Peripheral blood was collected from all individuals in heparin-containing tubes and peripheral blood mononuclear cells (PBMCs) from all collected blood samples were isolated by Ficoll-Paque density gradient centrifugation. PBMCs were cryopreserved in liquid nitrogen until analysis.

### S^+^ MBC quantification, culture and ELISPOT

Thawed PBMCs were first enriched for B cells using a pan B cell isolation kit (Miltenyi), according to the manufacturer’s guidelines. Biotinylated full-length Wuhan-Hu-1 S proteins (Miltenyi) were incubated with fluorescently labeled streptavidin (SA) for 15 min at room temperature^30,47–49^. Cells were stained with an antibody cocktail containing CD3, CD19, CD21, CD27, CD38, CD138, CD71, IgA, IgG, IgD, IgM and 7-AAD for 30 min at 4 °C before acquisition on the LSR Fortessa flow cytometer (BD). S^+^ MBCs were defined as live CD3^−^CD19^+^IgD^−^CD27^+^CD38^−/+^ S bispecific cells, whereas S^+^ plasmablasts were defined as live CD3^−^CD19^+^IgD^−^CD27^+^CD38^++^ S bispecific cells^31,48^. The gating strategy is shown in Supplementary Fig. 1. S^+^ MBC were isolated on the FACSAria III Cell Sorter (BD) and cultured in B cell expansion medium (Miltenyi), supplemented with 5% AB serum, 1% penicillin/streptomycin, IL-4, IL-21 and IL-2, at 37 °C for 7 days. Thereafter, cells were washed twice with IMDM + 10% ultralow IgG FCS before ELISPOT analysis. In brief, 96-well MultiScreen IP Filter Plates (Merck) were coated with full-length Wuhan-Hu-1 (Miltenyi), Delta (Sinobiological) or Omicron (Sinobiological) S protein at 10 μg ml^−1^ in PBS and incubated overnight at 4 °C. S^+^ MBC cells were incubated overnight in IMDM + 10% ultralow IgG FBS at 37 °C. Plates were washed twice and biotinylated goat anti-human IgG secondary antibody (Mabtech) added at 1 μg ml^−1^ in ELISPOT buffer (PBS + 0.5% ultralow IgG FCS) for 2 h. Then, 100 μl streptavidin-ALP (1:1,000) was added for 1 h before BCIP/NBT substrate addition. The plate was then air dried and imaged with a BD Immunospot Series 3B analyzer. Anti-human IgG monoclonal antibodies MT145 (Mabtech) were used as a positive control. R484 (2 μg ml^−1^) was used for additional stimulation. PBS was used as a negative control.

### Identification of asymptomatic SARS-CoV-2 infections

N-specific antibodies, anti-S IgG, as well as N-reactive T cell responses, were used to identify participants who were asymptomatically infected. N-reactive T cell responses were selected as a large proportion of SARS-CoV-2-convalescent individuals develop T cell responses against N and its absence among pre-pandemic donor samples demonstrates its low cross-reactivity with seasonal coronaviruses^9,11^. At each time point, any (1) newly positive anti-N antibodies, (2) fourfold rise in anti-S IgG in the absence of vaccination or (3) substantial increase in IFNγ or IL-2 were taken to represent an interim asymptomatic SARS-CoV-2 infection. A substantial increase in IFNγ or IL-2 was defined as either (1) a tenfold increase in cytokine levels from baseline, (2) a tenfold increase in cytokine levels from the last visit or (3) cytokine levels 10× above the threshold of positivity. As there is nonspecific T cell activation up to 4 weeks after vaccination, N-reactive T cell responses 10 days after dose one were not used for identification of asymptomatically infected individuals^8^.

### Statistics and reproducibility

All experiments were performed once with the number of individuals stated in the main manuscript and figure legends. The number of technical replicates for each assay is stated in Methods for the respective assay.

Statistical analyses, including the construction of ROC curves, were conducted using GraphPad Prism v.10.0.2. A two-tailed Mann–Whitney *U*-test was used for comparison of unpaired continuous data between two groups and a Kruskal–Wallis H test was used when more than two groups were compared. A Wilcoxon rank test was used for comparison of paired continuous data from the same individuals between different time points. A two-tailed Fisher’s exact test was used for comparison of categorical data between groups. A *P* < 0.05 level of confidence was accepted for statistical significance. The ROC curve analysis was performed using the Wilson/Brown test. A multivariate regression model was performed in R v.4.3.2 using packages stats v.4.3.2 and gtsummary v.1.7.2. Figures were created using GraphPad Prism v.10.0.2, FlowJo v.10.8.1 and BioRender. All box-and-whisker plots show the median (center line), interquartile range (box) and range (whiskers).

### Reporting summary

Further information on research design is available in the Nature Portfolio Reporting Summary linked to this article.

## Online content

Any methods, additional references, Nature Portfolio reporting summaries, source data, extended data, supplementary information, acknowledgements, peer review information; details of author contributions and competing interests; and statements of data and code availability are available at 10.1038/s41591-024-02962-3.

### Supplementary information

Supplementary Information Supplementary Fig. 1 and Table 1.

Reporting Summary

## Figures and Tables

**Extended Data Table 1:**
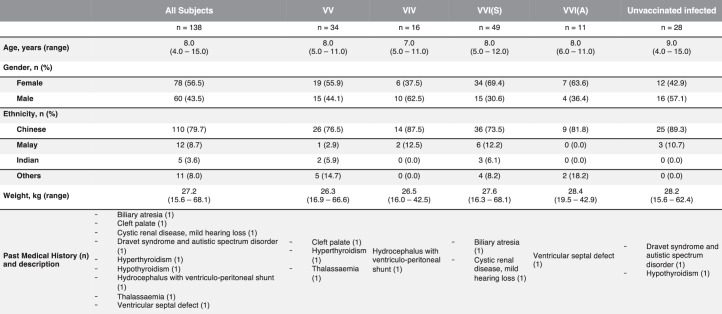
Baseline demographic and clinical features of all pediatric subjects

Subjects were broken down into groups: vaccine-only (VV), infection between 2 doses of vaccine (VIV), symptomatic infection after 2 doses of vaccine (VVI (S)), asymptomatic infection after 2 doses of vaccine (VVI (A)), and unvaccinated infected. Fisher’s Exact test was done to compare demographic features between groups, using ‘All subjects’ as the reference; *: p < 0.05.

**Extended Data Table 2:**
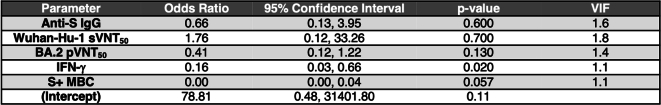
Multivariate regression model of the humoral and cellular immune parameters and their correlation with symptomatic SARS-CoV-2 infection between 3 and 6 months post-vaccination (dependent variable)

Note that while immunological parameters cannot be truly considered to be independent variables for a multivariate regression model, Variance Inflation Factors (VIF) of less than 5 between a given predictor variable and other predictor variables in the model are usually not severe enough to invalidate the model.

## Data Availability

All aggregate data supporting the findings of this study are available within the paper and its supplementary materials. Individual-level participant data are not publicly available. Only the data of individuals who consented to further research can be accessible with the consent of the ethics committees from the requestor’s and corresponding authors’ institutions. A formal data transfer agreement between the institutions will be required upon ethics approval. The corresponding authors can be contacted for access to data and will respond within 1 month; data transfer can take place once the data transfer agreement is completed.
