# Publisher Correction: Correlates of protection against symptomatic SARS-CoV-2 in vaccinated children

**DOI:** 10.1038/s41591-024-03107-2

**Published:** 2024-06-10

**Authors:** Youjia Zhong, Alicia Y. H. Kang, Carina J. X. Tay, Hui’ En Li, Nurul Elyana, Chee Wah Tan, Wee Chee Yap, Joey M. E. Lim, Nina Le Bert, Kuan Rong Chan, Eugenia Z. Ong, Jenny G. Low, Lynette P. Shek, Elizabeth Huiwen Tham, Eng Eong Ooi

**Affiliations:** 1 Department of Paediatrics, Yong Loo Lin School of Medicine, National University of Singapore (NUS), Singapore, Singapore; 2 Programme in Emerging Infectious Diseases, Duke-NUS Medical School, Singapore, Singapore; 3 Khoo Teck Puat-National University Children’s Medical Institute, National University Health System (NUHS), Singapore, Singapore; 4 Infectious Diseases Translational Research Programme, Department of Microbiology and Immunology, Yong Loo Lin School of Medicine, National University of Singapore, Singapore, Singapore; 5 Viral Research and Experimental Medicine Centre, SingHealth Duke-NUS Academic Medical Centre, Singapore, Singapore; 6 Department of Infectious Diseases, Singapore General Hospital, Singapore, Singapore; 7 Department of Clinical Translational Research, Singapore General Hospital, Singapore, Singapore

**Keywords:** Adaptive immunity, Infectious diseases

Correction to: *Nature Medicine* 10.1038/s41591-024-02962-3, published online 30 April 2024

This paper was originally published under standard Springer Nature license (© The Author(s), under exclusive licence to Springer Nature America, Inc.). It is now available as an Open Access paper under a Creative Commons Attribution 4.0 International license, © The Author(s). The error has been corrected in the HTML and PDF versions of the article.

