# Supplementary material for: Correlates of protection against symptomatic SARS-CoV-2 in vaccinated children
Source: Nat Med. 2024 Apr 30;30(5):1373–83. doi: 10.1038/s41591-024-02962-3 (PMC11164684; doi:10.1038/s41591-024-02962-3)
Supplement: Supplementary file 1 — Supplementary Fig. 1 and Table 1. [file 41591_2024_2962_MOESM1_ESM.pdf]

nature medicine

Article https://doi.org/10.1038/s41591-024-02962-3

Correlates of protection against symptomatic SARS-CoV-2 in vaccinated children

In the format provided by the authors and unedited

Longitudinal adaptive immune profiling of vaccinated children reveals correlates of protection against symptomatic SARS-CoV-2

Supplementary Information

Supplementary Figure 1. Flow cytometric gating strategy for Spike-specific memory B cells (S+ MBC), for both S+ MBC quantification, and B cell ELISPOT.

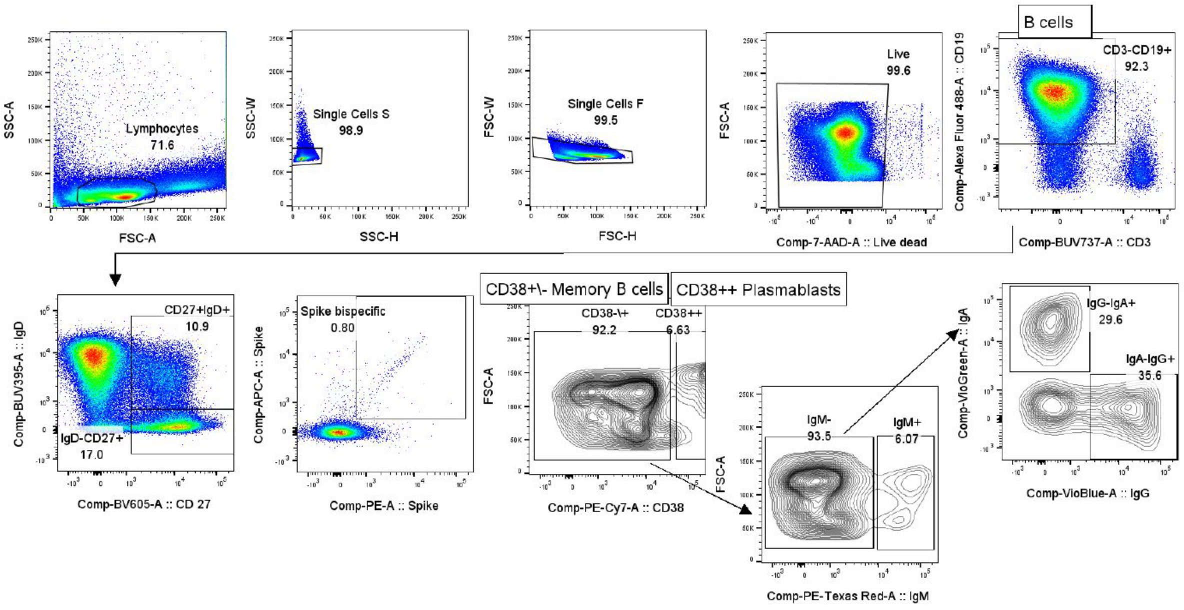

Supplementary Table 1: P-values when immunological parameters of children with vaccine-only immunity were tested against demographic features age, weight, gender and ethnicity. **A.** Immunological parameters induced by vaccine-only at 3 months (n=83), and **B.** at 6 months (n=34).

A

Demographic feature	Anti-Spike IgG at 3 months	$SVNT_{50}$ titres against Wuhan-Hu-1 at 3 months	Spike+ MBCs at 3 months	Interferon-γ at 3 months	Interleukin-2 at 3 months	Statistical test used
Age	0.42	0.53	0.85	0.53	0.48	Spearman correlation
Weight	0.95	0.39	0.93	0.85	0.36	Spearman correlation
Gender	0.15	0.07	0.74	0.14	0.25	Two-tailed Mann Whitney U
Ethnicity	0.21	0.73	0.91	0.90	0.77	One-way ANOVA

B

Demographic feature	Anti-Spike IgG at 6 months	$SVNT_{50}$ titres against Wuhan-Hu-1 at 6 months	Spike+ MBCs at 6 months	Interferon-γ at 6 months	Interleukin-2 at 6 months	Statistical test used
Age	0.45	0.46	0.96	0.46	0.07	Spearman correlation
Weight	0.49	0.62	0.75	0.88	0.21	Spearman correlation
Gender	0.38	0.41	0.60	0.98	0.70	Two-tailed Mann Whitney U
Ethnicity	0.66	0.63	0.79	0.39	0.83	One-way ANOVA
