# Supplementary material for: Correlates of protection against symptomatic SARS-CoV-2 in vaccinated children
Source: Nat Med. 2024 Apr 30;30(5):1373–83. doi: 10.1038/s41591-024-02962-3 (PMC11164684; doi:10.1038/s41591-024-02962-3)
Supplement: Supplementary file 2 — Reporting Summary [file 41591_2024_2962_MOESM2_ESM.pdf]

nature portfolio

Corresponding author(s): Eng Eong Ooi, Youjia Zhong

Last updated by author(s): Mar 25, 2024

Reporting Summary

Nature Portfolio wishes to improve the reproducibility of the work that we publish. This form provides structure for consistency and transparency in reporting. For further information on Nature Portfolio policies, see our Editorial Policies and the Editorial Policy Checklist.

Statistics

For all statistical analyses, confirm that the following items are present in the figure legend, table legend, main text, or Methods section.

n/a	Confirmed	
☐	☒	The exact sample size (*n*) for each experimental group/condition, given as a discrete number and unit of measurement
☒	☐	A statement on whether measurements were taken from distinct samples or whether the same sample was measured repeatedly
☐	☒	The statistical test(s) used AND whether they are one- or two-sided<br>*Only common tests should be described solely by name; describe more complex techniques in the Methods section.*
☒	☐	A description of all covariates tested
☒	☐	A description of any assumptions or corrections, such as tests of normality and adjustment for multiple comparisons
☐	☒	A full description of the statistical parameters including central tendency (e.g. means) or other basic estimates (e.g. regression coefficient) AND variation (e.g. standard deviation) or associated estimates of uncertainty (e.g. confidence intervals)
☐	☒	For null hypothesis testing, the test statistic (e.g. *F*, *t*, *r*) with confidence intervals, effect sizes, degrees of freedom and *P* value noted<br>*Give P values as exact values whenever suitable.*
☒	☐	For Bayesian analysis, information on the choice of priors and Markov chain Monte Carlo settings
☒	☐	For hierarchical and complex designs, identification of the appropriate level for tests and full reporting of outcomes
☒	☐	Estimates of effect sizes (e.g. Cohen's *d*, Pearson's *r*), indicating how they were calculated

Our web collection on statistics for biologists contains articles on many of the points above.

Software and code

Policy information about availability of computer code

Data collection	Clinical data was collected and stored in commercially available REDCap 14.2.2 and exported for analysis in Microsoft Excel. Python 3.9.2 was used to combine immunological parameters and clinical data.
Data analysis	GraphPad Prism 10.0.2 was used for most of the data analysis, including box and whiskers plots, tow-tailed Mann Whitney-U tests, Wilcoxon Rank tests, two-tailed Fisher exact test, ROC curves with Wilson/Brown tests, and bar charts. UMAP for 8-cytokine release assay was generated using R version 4.3.0, incorporated as additional parameters and converted to FCS files and loaded into FlowJo to generate heatmaps. Multivariate regression model was done in R 4.3.2, using packages stats 4.3.2 and gtsummary 1.7.2. Biorender was used to create schematics.

For manuscripts utilizing custom algorithms or software that are central to the research but not yet described in published literature, software must be made available to editors and reviewers. We strongly encourage code deposition in a community repository (e.g. GitHub). See the Nature Portfolio guidelines for submitting code & software for further information.

Data

Policy information about availability of data

All manuscripts must include a data availability statement. This statement should provide the following information, where applicable:

- Accession codes, unique identifiers, or web links for publicly available datasets
- A description of any restrictions on data availability
- For clinical datasets or third party data, please ensure that the statement adheres to our policy

All aggregate data supporting the findings of this study are available within the paper and its supplementary materials. Individual-level subject data are not publicly available. Only the data of subjects who consented to further research can be accessible with the consent of the ethics committees from the requestor's and corresponding author's institutions. A formal data transfer agreement (DTA) between the institutions will be required upon ethics approval.

Research involving human participants, their data, or biological material

Policy information about studies with human participants or human data. See also policy information about sex, gender (identity/presentation), and sexual orientation and race, ethnicity and racism.

Reporting on sex and gender	Sex was not considered for enrollment, and is reported in aggregate here based on extraction from parent or subject report.
Reporting on race, ethnicity, or other socially relevant groupings	Race and ethnicity were not considered for enrollment, and is reported in aggregate here based on extraction from parent or subject report.
Population characteristics	The study involving paediatric subjects took place in the National University Hospital, a tertiary academic medical centre in Singapore. Healthy children aged 5 to 12 years old, eligible for BNT162b2 vaccination, were recruited from the general population between 20 December 2021 and 8 March 2022.<br><br>The study involving adult subjects took place in the Singapore General Hospital, also a tertiary academic medical centre in Singapore. Healthy healthcare workers aged 25-89 years old, eligible for BNT162b2 vaccination, were recruited from the hospital between 5 January 2021 and 8 September 2021.
Recruitment	Children were recruited through advertisements around the National University Hospital, Singapore and in the community, with written parental consent and written subject assent. As COVID-19 vaccination was voluntary, this method of recruitment could potentially self-select for families which have higher anxiety about the pandemic, which are likely to employ non-pharmacological methods to avoid infection. Thus, the number of children who were infected with SARS-CoV-2 may be lower in our cohort than the general population; this enabled us to study vaccine-only immunity in children.<br><br>Eligible adult participants were approached for recruitment by the study team members. As healthcare workers may be more aware and diligent with non-pharmacological methods of preventing COVID-19, the infection rate of these adults may be lower than that of the general population. This is not likely to have a significant impact on our results.
Ethics oversight	The study protocols for the paediatric cohorts were approved by the National Healthcare Group Domain Specific Review Board (NHG DSRB) (2021/00945, 2021/00984, 2022.00316). The study protocol for the adult cohort was approved by the SingHealth Centralised Institutional Review Board (CIRB/F 2021/2024).

Note that full information on the approval of the study protocol must also be provided in the manuscript.

Field-specific reporting

Please select the one below that is the best fit for your research. If you are not sure, read the appropriate sections before making your selection.

☒ Life sciences ☐ Behavioural & social sciences ☐ Ecological, evolutionary & environmental sciences

For a reference copy of the document with all sections, see nature.com/documents/nr-reporting-summary-flat.pdf

Life sciences study design

All studies must disclose on these points even when the disclosure is negative.

Sample size	As the study was originally designed to study immunogenicity of mRNA vaccines in children, we aimed to recruit 100 children based on numbers of subjects in other published studies, and taking into account subject attrition rate. As the Omicron variant emerged after study initiation, providing a fortuitous opportunity for us to identify immune correlates of protection, there was no a priori power calculation performed for the identification of correlates of protection.
Data exclusions	No data was excluded.
Replication	This natural experiment of primary vaccination of children during the SARS-CoV-2 pandemic could not be replicated.

Randomization	Randomisation was not relevant to our study, as all subjects received the same vaccine.
Blinding	Blinding was not relevant to our study, as all subjects received the same vaccine.

Reporting for specific materials, systems and methods

We require information from authors about some types of materials, experimental systems and methods used in many studies. Here, indicate whether each material, system or method listed is relevant to your study. If you are not sure if a list item applies to your research, read the appropriate section before selecting a response.

Materials & experimental systems

n/a	Involved in the study
☐	☒ Antibodies
☐	☒ Eukaryotic cell lines
☒	☐ Palaeontology and archaeology
☒	☐ Animals and other organisms
☒	☐ Clinical data
☒	☐ Dual use research of concern
☒	☐ Plants

Methods

n/a	Involved in the study
☒	☐ ChIP-seq
☐	☒ Flow cytometry
☒	☐ MRI-based neuroimaging

Antibodies

Antibodies used

Anti-S IgG:
1. Anti-SARS-CoV-2 RBD neutralising antibodies
Acrobiosystems, Cat number SAD-S35. Diluted 633x
2. HRP-IgG secondary antibody
Life technologies, Cat number 31413, lot number 081112I. Diluted 1000x

pVNT:
1. Anti-VZV-G mAb
2. Kerafast, Cat Number EB0010, Lot220104, dilution 1:5000

MBC ELISPOT:
1. Biotinylated goat anti-human IgG secondary antibody
Mabtech, Cat number 3820-4-250, polyclonal, lot number: batch 4.2
Dilution 1mcg/ml
2. Anti-human IgG mAb MT145
Mabtech, Cat number 3850-1-250, clone name MT145, lot number: batch 9.1
Dilution 15mcg/ml

Validation

Validation information (just need the website for the product) needed for:
1. Anti-SARS-CoV-2 RBD neutralising antibodies

2. Anti-VZV-G mAb

3. Anti-human IgG mAb MT145

All antibodies were commercially available and validated by the manufacturers with details available on the manufacturer's websites and / or referenced as follows:
https://jp.acrobiosystems.com/P3209-Anti-SARS-CoV-2-Spike-RBD-Neutralizing-Antibody-Human-IgG1-%28AS35%29.html
https://www.kerafast.com/productgroup/1009/anti-vsv-g-8g5f11-antibody?ProductID=444
https://www.mabtech.com/products/goat-anti-human-igg-biotin-3820-4
https://www.mabtech.com/products/anti-human-igg-mab-mt145-unconjugated-3850-1

Eukaryotic cell lines

Policy information about cell lines and Sex and Gender in Research

Cell line source(s)	A549-ACE2 cells were produced by transduction of advanced-generation lentiviruses carrying human ACE2 gene under EF1-alpha promoter in pFUGW vector. A549-ACE2 cells were maintained in RPMI-1640 supplemented with 10% FBS and 15 µg ml −1 blasticidin.
Authentication	NA
Mycoplasma contamination	None

Commonly misidentified lines
(See ICLAC register)

NA

Flow Cytometry

Plots

Confirm that:

☒ The axis labels state the marker and fluorochrome used (e.g. CD4-FITC).

☒ The axis scales are clearly visible. Include numbers along axes only for bottom left plot of group (a 'group' is an analysis of identical markers).

☒ All plots are contour plots with outliers or pseudocolor plots.

☒ A numerical value for number of cells or percentage (with statistics) is provided.

Methodology

Sample preparation

Thawed PBMCs were first enriched for B cells using pan B cell isolation kit (Miltenyi, Germany), according to manufacturer's guidelines. Biotinylated full-length Wuhan-Hu-1 S proteins (Miltenyi, Germany) were incubated with fluorescently labeled streptavidin (SA) for 15 minutes at room temperature.. Cells were stained with an antibody cocktail containing CD3, CD19, CD21, CD27, CD38, CD138, CD71, IgA, IgG, IgD, IgM and 7-AAD for 30minutes at 4°C prior to acquisition.

Instrument

BD LSR Fortessa

Software

Flowjo 10.8.1

Cell population abundance

Final population of interest was 0.001% - 1% of total B cells.

Gating strategy

CD3-CD19+IgD-CD27+CD38-/+S bispecific cells were identified using gating strategy in Supplementary figure 1.

☒ Tick this box to confirm that a figure exemplifying the gating strategy is provided in the Supplementary Information.
